# Callus organoids reveal distinct cartilage to bone transition mechanisms across donors and a role for biological sex

**DOI:** 10.1038/s41413-025-00418-z

**Published:** 2025-03-26

**Authors:** Isaak Decoene, Hanna Svitina, Mohamed Belal Hamed, Anastassios Economou, Steve Stegen, Frank P. Luyten, Ioannis Papantoniou

**Affiliations:** 1 Skeletal Biology and Engineering Research Center, Department of Development and Regeneration, KU Leuven, O&N1, Herestraat 49, box 813, 3000 Leuven, Belgium; 2 Prometheus Translational Division of Skeletal Tissue Engineering, KU Leuven, O&N1, Herestraat 49, box 813, 3000 Leuven, Belgium; 3 Laboratory of Molecular Bacteriology, Department of Microbiology, Immunology and Transplantation, Rega Institute, KU Leuven, Herestraat 49, 3000 Leuven, Belgium; 4 Molecular Biology Department, National Research Centre, 33 El Buhouth st, Dokii 12622 Cairo, Egypt; 5 Department of Neurosciences, Leuven Research Institute for Neuroscience and Disease (LIND), KU Leuven, VIB-KU Leuven Center for Brain & Disease Research, Herestraat, 3000 Leuven, Belgium; 6 Laboratory of Clinical and Experimental Endocrinology, Department of Chronic Diseases and Metabolism (CHROMETA), KU Leuven, Herestraat 49, 3000 Leuven, Belgium; 7 Institute for Chemical Engineering Sciences, Foundation for Research and Technology–Hellas (FORTH), Stadiou Street, Platani, box 1414, 26504 Patras, Greece

**Keywords:** Bone, Bone quality and biomechanics

## Abstract

Clinical translation of tissue-engineered advanced therapeutic medicinal products is hindered by a lack of patient-dependent and independent in-process biological quality controls that are reflective of in vivo outcomes. Recent insights into the mechanism of native bone repair highlight a robust path dependence. Organoid-based bottom-up developmental engineering mimics this path-dependence to design personalized living implants scaffold-free, with in-build outcome predictability. Yet, adequate (noninvasive) quality metrics of engineered tissues are lacking. Moreover, insufficient insight into the role of donor variability and biological sex as influencing factors for the mechanism toward bone repair hinders the implementation of such protocols for personalized bone implants. Here, male and female bone-forming organoids were compared to non-bone-forming organoids regarding their extracellular matrix composition, transcriptome, and secreted proteome signatures to directly link in vivo outcomes to quality metrics. As a result, donor variability in bone-forming callus organoids pointed towards two distinct pathways to bone, through either a hypertrophic cartilage or a fibrocartilaginous template. The followed pathway was determined early, as a biological sex-dependent activation of distinct progenitor populations. Independent of donor or biological sex, a cartilage-to-bone transition was driven by a common panel of secreted factors that played a role in extracellular matrix remodeling, mineralization, and attraction of vasculature. Hence, the secreted proteome is a source of noninvasive biomarkers that report on biological potency and could be the missing link toward data-driven decision-making in organoid-based bone tissue engineering.

## Introduction

The field of skeletal tissue engineering continues to evolve towards a stage where it can meet the high expectations set during the previous decade. However, there are still limited examples of cell-based skeletal tissue engineering strategies that have been successfully translated into the clinic,^1^ which can be attributed mostly to scaling and manufacturing challenges. In recent years, researchers have incorporated manufacturing and regulatory principles early on in the development cycle.^2^ Still, a major hurdle towards clinical translation of autologous skeletal tissue-engineered implants is a lack of robust quality controls that can predict the implant regenerative capacity and potency. Moreover, there is a lack of metrics that reflect biological variability and which can be measured noninvasively to enable closed-system manufacturing and in-process decision-making.^3–6^

A leading paradigm in Tissue Engineering is the emergence of bottom-up approaches whereby functional organoid populations are used as building blocks for producing larger meso- and macro-tissue structures.^7^ These populations provide a scalable approach that, due to their relative individual simplicity and defined size, enables the generation of more controlled tissues in vitro.^8^ Such bottom-up formats have enabled the implementation of ‘developmental engineering’ strategies, that is, the recapitulation of robust biological cascades encountered in developmental systems such as embryonic growth of the appendicular skeleton or fracture repair through endochondral ossification.^9,10^ Herein, bone is formed by gradual remodeling and mineralization of a soft cartilage intermediate tissue.

During endochondral bone development, the cartilage intermediate is produced solely by chondroprogenitors undergoing hypertrophic differentiation. However, postnatal fracture repair is more complex with varying patient- and situation-dependent systemic factors such as biological sex, age and disease,^11,12^ environmental contaminations, immune system,^13^ mechanical stimuli, and cellular communication.^14^ These systemic factors are crucial in the early inflammatory phase and the formation of a fracture hematoma.^15^ Bicortical bone repair requires the activation of progenitor cells which reside in the periosteum, the fibrous outer layer of long bones.^16^ Progenitors from the periosteum then create a cartilaginous soft callus which then interacts with the host vascular and immune system towards the formation of a mineralized cartilaginous callus followed by complete remodeling into structured and mechanically robust bone tissue.^17^ The role of systemic factors, such as immune cell activity, biological sex, and age have been well studied for both the early phase and the late remodeling phase,^18,19^ but little is known about the factors that influence the soft callus composition and kinetics.

Periosteal fracture repair is translated as developmental blueprint for the design of skeletal tissue-engineered products through the formation of a cartilaginous callus intermediate. This intermediate is mineralized by sequentially activating biological pathways which are regulated by secreted proteins and soluble signaling molecules.^20–22^ This approach has been successfully demonstrated via the use of human periosteal callus organoid-based tissues able to form bone ossicles ectopically and repair bone defects upon implantation in murine models.^23,24^ Yet, the use of callus organoids for bone tissue engineering raises the question on the roles of biological variability between human patients and how this relates to the insights gained in mice.^25^ Clinical data focus mostly on the effect of age or biological sex in an aging population which is dominated by postmenopausal changes in hormone levels.^26^ However, the peak incidence of bone fractures and the population primarily requiring tissue-engineered implants for delayed or non-unions, centers around patients aged between 25 and 45 years.^27^ Hence, a need arises to understand factors like health background and biological sex in the context of in vitro models, also in younger patients.^28^ Indeed, efforts to define predictive markers of mesenchymal stromal cell (MSC) chondrogenic potential have struggled with the extent of biological variation.^29^ Still, to engineer bone implants, in particular personalized autologous ones, a complete understanding of biological variation must be coupled to measurable potency assays linked to in vivo outcomes.^30^

Developmental cascades show tight regulation and robustness. Hence, a robust panel of metrics can be defined by precisely measuring quality attributes during developmental engineering processes. By linking measurable quality attributes to in vivo outcomes, critical quality attributes (CQA) as markers of tissue implant functionality can be identified. This capacity is key for clinical translation through the integration of Quality by design (QbD) principles.^31^ These principles are also reflected in the regulatory guidelines of the European Medicines Agency (EMA) and the Committee for Advanced Therapies (CAT). These require detailed characterization of the implanted construct including efficacy endpoint markers linked to its therapeutic efficacy or bone-forming capacity after implantation.^32,33^ However, universal markers that may be reflective of the chondrogenic differentiation process for tissue engineered products, irrespective of donor properties but predictively linked to robust in vivo potency, are currently lacking. In addition, tissue engineered products are required to provide data on safety, quality, and efficacy before they are implanted. This leads to additional sampling of patient cells which is both costly and unnecessarily invasive.^30,34^ Moreover, in-process sampling for quality measurements is invasive and increases the risk for contamination and errors. Non-invasive measurements, on the other hand, avoid additional sampling and manual interventions, thus facilitating closed-system manufacturing.

In this study, we generated high volumes of donor-specific homogenous callus organoid populations to create donor-specific implants through bio-assembly. We carried out a series of orthogonal quality characterization studies and used both organoid transcriptome and sample-matched high-sensitivity proteomics for mapping the secretome during chondrogenic differentiation of callus organoids in xeno-free differentiation medium.

## Results

### Donor-derived callus organoids as building blocks for developmental tissue engineering

**Fig. 1:**
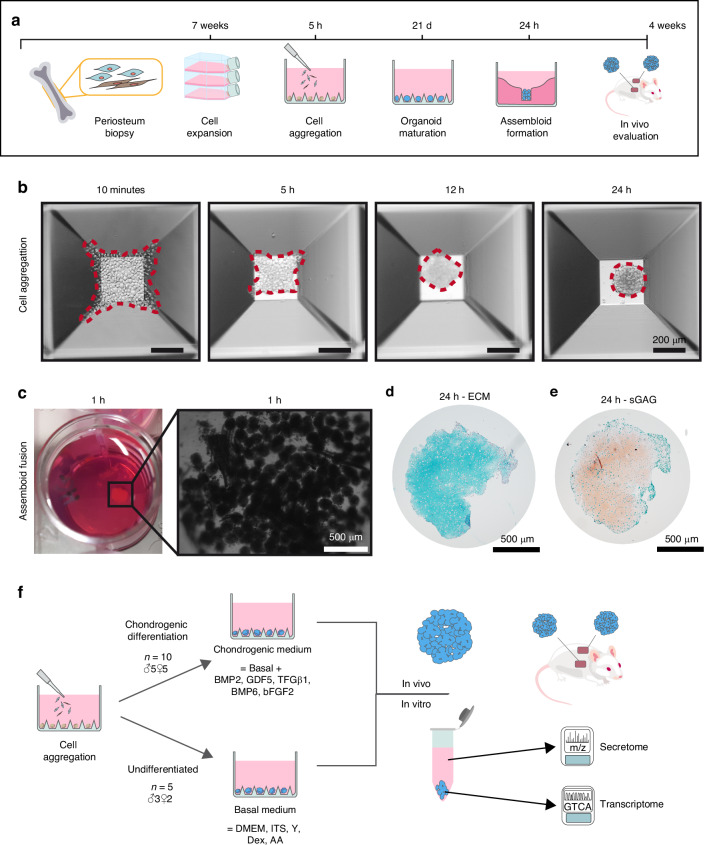
Experimental design and methods overview. **a** Protocol timeline. **b** The process of cell aggregation and organoid formation. The dotted line represents the organoid edge. Scalebar = 200 µm. **c** organoid fusion leading to the formation of macro-scale assembloids. **d** Alcian blue staining of cartilaginous extracellular matrix (ECM) of an assembloid after 24 h, and (**e**) safranin-o/fastgreen staining of sulphated glycosaminoglycans (sGAG). Scalebar = 500 µm. **f** Experimental design to assess the requirements for cartilage-to-bone transition. For chondrogenic treatment, cells from 10 donors were evaluated (5 male, 5 female, aged 16–29). As undifferentiated control, 5 donors were evaluated (3 male, 2 female, age 21–29)

Bottom-up tissue engineering of bone-forming callus organoids is illustrated in Fig. 1a. Human periosteum-derived cells (hPDCs) were drop seeded in an aggrewell800 nonadherent microwell platform. Figure 1b shows the spontaneous cell aggregation process through gravitational force when cells are seeded into a non-adherent microwell.

Chondrogenic to pre-hypertrophic differentiation was done by culturing the organoids in a xenofree, low-protein differentiation medium previously developed^35^ and characterized.^36^ Here, periosteal progenitor cells are activated, following a proliferation phase and chondrogenic differentiation phase with the deposition of cartilaginous extracellular matrix during the first week. Then, between 14 and 21 days, a maturation of the phenotype occurs, followed by a catabolic phase characterized by pre-hypertrophic differentiation and extracellular matrix remodeling. During this phase, organoids progressively show a decreased ability to undergo bio-assembly^37^ and signs of terminal differentiation.^38^

Using this strategy, within 5 h the cells aggregated at the bottom of the microwell, forming an early callus organoid. These organoids underwent chondrogenic differentiation for 21 days, after which 600 organoids were collected in an agarose mold for assembloid formation as shown in Fig. 1c. The assembloid was cultured in chondrogenic medium for an additional 24 h to allow spontaneous tissue fusion into a coherent cartilage implant that was characterized by a cartilaginous extracellular matrix (ECM) and deposition of sulphated glycosaminoglycans (sGAGs) as shown in Fig. 1d–e. These cartilaginous tissue intermediates were then implanted ectopically in vivo in nude mice for 4 weeks to allow host tissue cell invasion and cartilage-to-bone transition.

To investigate the role of cell identity, extracellular matrix composition, and paracrine signaling as potential drivers of cartilage-to-bone transition, hPDCs were collected from 10 human donors (*n* = 10, 5 male, 5 female, aged 16–29). As illustrated in Fig. 1f, donor-derived callus organoids were created in culture conditions directing chondrogenic differentiation. In parallel, control organoids (*n* = 5, 3 male, 2 female, aged 21–29) also underwent cellular aggregation and 3D culture, but not cultured in chondrogenic medium. After 21 days, *n* = 4 assembloids were created per donor and implanted ectopically in nude mice to assess bone-forming potency of donor-derived implants. At this time, organoids were collected together with their conditioned medium for histological analysis of tissue morphology, transcriptome analysis, and secreted proteome analysis.

### Early proliferation determines callus organoid size kinetics and morphology

**Fig. 2:**
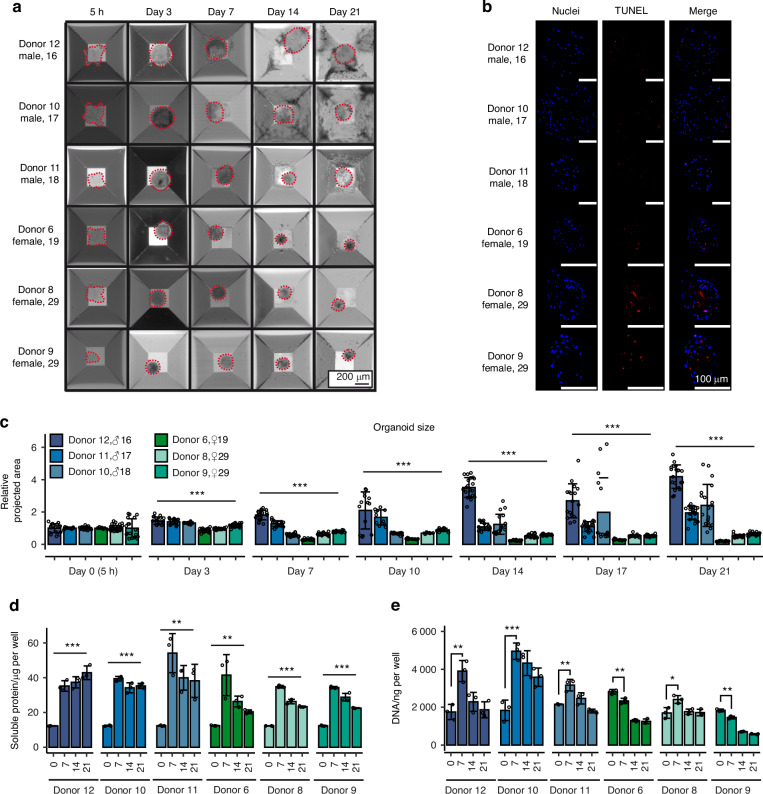
Timeseries analysis of male and female donor-derived organoid populations. **a** Brightfield imaging of chondrogenic treated callus organoids. The dotted line represents the organoid edge. Scalebar = 200 µm. **b** Apoptosis staining of callus organoids. Scale bar = 100 µm. **c** Size quantification of callus organoids over time (*n* = 18) representative organoids per donor. **d** Total amount of soluble proteins in conditioned medium over time (*n* = 3). **e** DNA evolution during chondrogenic differentiation (*n* = 3). Data is represented as mean ± s.d. *P < 0.05, **P < 0.01, ***P < 0.001

Callus organoids were created through a process of self-assembly and chondrogenic differentiation of periosteal cells derived from 6 donors (3 male, 3 female, aged 16–29). Time-lapse brightfield imaging (Fig. 2a) shows the self-assembly process and initial cell aggregate formation during the first 5 h, followed by 21 days of differentiation. During chondrogenic differentiation, we observed two groups with distinct organoid morphology. One group of donor organoids was more transparent and strongly increased in size over time. In contrast, the second group also formed organoids successfully within 5 h, but their size did not increase over time, and the organoids were more optically dense. TUNEL staining, shown in Fig. 2b, confirmed high cell viability for all organoids at day 21 of differentiation. In Fig. 2c, morphological analysis of brightfield images allowed us to track organoid size over time. Here we observed a gradual distinction between the male and female organoids within the first week of differentiation. 2 male donors generated organoids which showed a significant size increase already after 3 days, while the third male donor displayed a size increase after 14 days. In contrast, the female donor organoid populations had a more uniform size distribution, but their organoid size dropped after three days, remaining unchanged up to day 21. Despite these morphological differences, both male and female organoid populations produced similar amounts of soluble protein (Fig. 2d), which peaked in the first 7 days. Interestingly, the cell number evolution, as indirectly assessed via DNA quantification (Fig. 2e), also distinguished male versus female organoid populations based on their increase in cell number. Male exhibiting an enhanced increase in cell number during the first 7 days, and continued to grow in size also when cell numbers remained unchanged, likely through the synthesis and deposition of extracellular matrix. Female organoids on the other hand, underwent only a limited or no initial increase in cell number and hence did not increase in size.

### Callus organoids display donor-dependent hypertrophic cartilage or fibrocartilage phenotypes

**Fig. 3:**
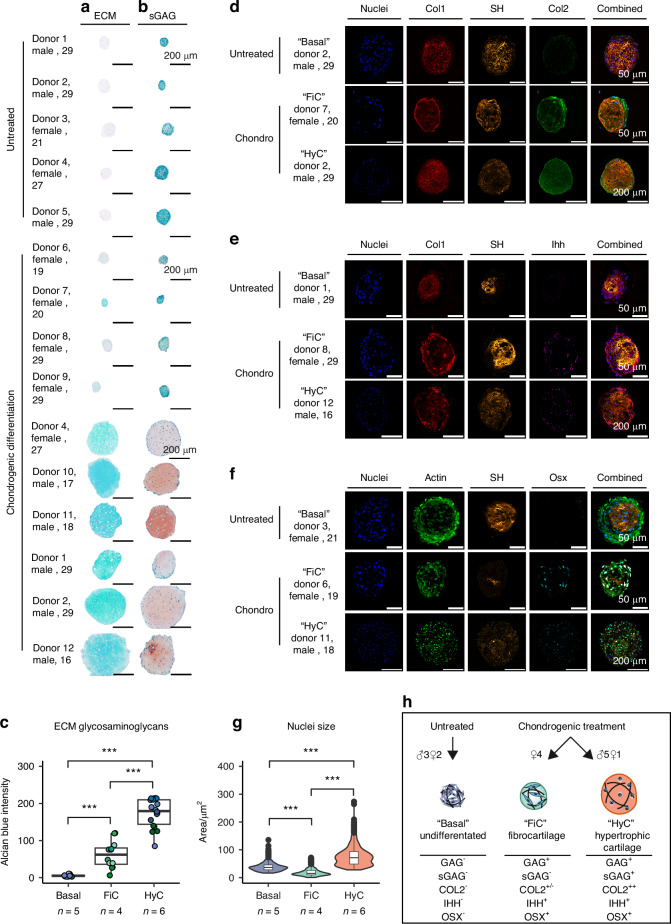
Histological analysis of day 21 organoids. **a** glycosaminoglycan extracellular matrix (ECM) and **b** sulphated glycosaminoglycan extracellular matrix (sGAG). Scalebar = 50 µm/200 µm as indicated. **c** Quantitative extracellular matrix intensity. **d** Immunostaining showing representative organoids stained for nuclei, collagen 1 (Col1), structured collagen through second harmonic generation imaging (SH), collagen 2 (Col2). **e** Nuclei, collagen 1, structured collagen, Indian hedgehog (Ihh). **f** Nuclei, actin, structured collagen, osterix (Osx). Scalebar = 50 µm or 200 µm as indicated. **g** Nucleus size quantification. **h** Summary of histological characteristics for undifferentiated (basal), fibrocartilaginous (FiC), and hypertrophic cartilage (HyC) organoids

To further assess the impact of biological sex on chondrogenic differentiation and the formation of functional callus organoids, additional biological replicates were evaluated, starting from a tissue-level characterization of the callus organoids. Therefore, as illustrated in Fig. 1f, we created callus organoids using hPDCs from 10 donors (*n* = 10, 5 male, 5 female, aged 16–29) through a 21-day chondrogenic differentiation and compared those to undifferentiated organoids (*n* = 5, 3 male, 2 female, aged 21–29) as negative control. Figure 3a shows a representative organoid for each donor and condition, stained for cartilaginous extracellular matrix (ECM), and in Fig. 3b sulphated glycosaminoglycans (sGAG) which is a more mature cartilage indicator. These histological stainings confirmed the significant size differences observed between callus organoids derived from different donors, where smaller callus organoids were of similar size as the negative undifferentiated control. However, histological staining and quantification in Fig. 3c showed a positive staining of cartilaginous extracellular matrix in all organoids undergoing chondrogenic differentiation, but not in the undifferentiated controls. Furthermore, only the large organoids stained positive for sGAGs in Fig. 3b and displayed hypertrophic cartilage morphology including enlarged hypertrophic cells. Next, immunohistochemical labeling was performed for cartilage-specific extracellular matrix protein collagen II (Col II). This confirmed the deposition of collagen II in all organoids following chondrogenic differentiation, but not in undifferentiated controls. In addition, immunohistochemical labeling of general extracellular matrix protein collagen I (Col I) in combination with second harmonic generation imaging showed a high level of structurally organized Col I positive matrix, mostly at the core of the organoids, including the undifferentiated controls. Moreover, as shown in Fig. 3e–f, chondrogenic stimulation was sufficient to obtain cells that are positive for the prehypertrophic marker, Indian hedgehog (Ihh), as well as the late hypertrophic/osteogenic marker Osterix (Osx). Therefore, we termed this phenotype as hypertrophic cartilage (HyC). As Fig. 3d–f shows one representative organoid for each group, Figs. S1–3 contain extended datasets for all donors. In these stainings, we observed notable differences in cell morphology, density, and nucleus size. The latter was quantified in Fig. 3g, showing that while large hypertrophic organoids also had an increased nucleus size, smaller organoids were made up of cells that were on average smaller than that of the cell population in undifferentiated organoids. As these stain positive for cartilage ECM, but are characterized by high cell density and fibrous cell morphology, we termed this phenotype as fibrocartilage (FiC). Taken together, and summarized in Fig. 3h, we observed two distinct responses to chondrogenic stimulation: one set of donors (*n* = 6, 5 males and 1 female) generates large hypertrophic cartilage callus organoids (HyC) positive for high volume ECM, sGAGs, Col II, Ihh and Osx; and a second set of female donors (*n* = 4) with a FiC phenotype which displayed signs of chondrogenic differentiation and ECM deposition, but a lacked ECM volume, ECM maturation and hypertrophic cellular morphology.

### Transcriptome analysis reveals a common chondrogenic identity between callus organoids

**Fig. 4:**
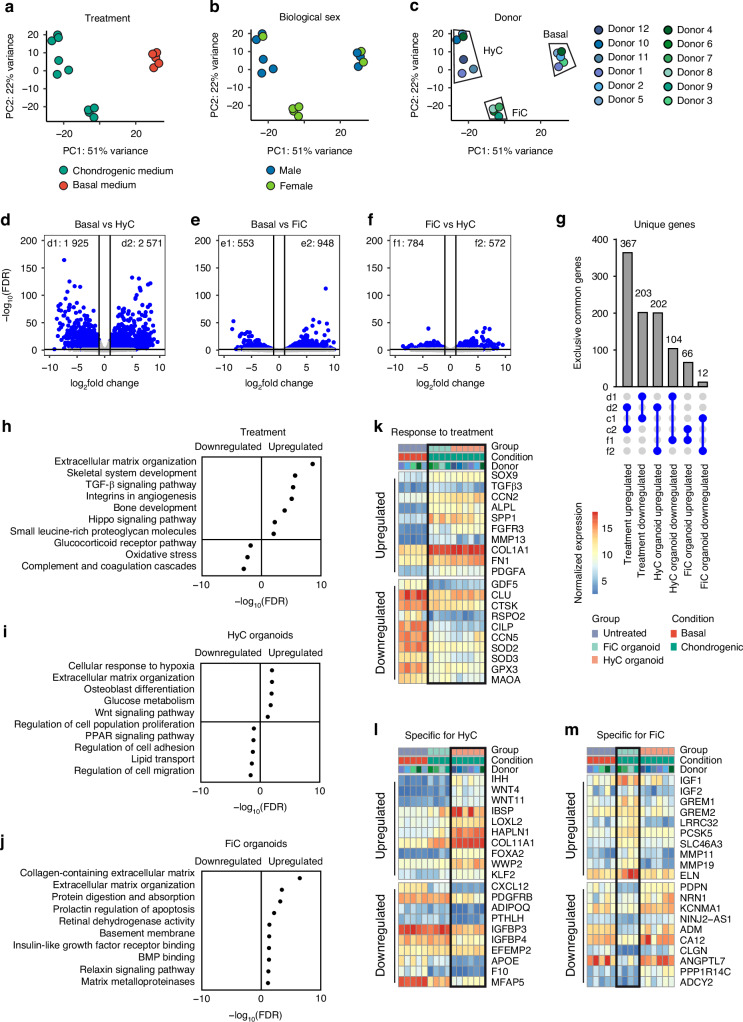
Transcriptome analysis of day 21 organoids. Principal component analysis colored by (**a**) experimental condition, (**b**) donor biological sex, and (**c**) donor. **d**–**f** Differential expression analysis showing the amount of differentially expressed genes (DEGs) per comparison. **g** Gene panel selection criteria. **h**–**j** Gene ontology summary for genes specific to chondrogenic treatment, hypertrophic organoids, or fibrocartilaginous organoids. **k**–**m** Gene panel selections for each organoid type shown as normalized expression. Abbreviations: hypertrophic cartilage organoid (HyC), fibrocartilage organoid (FiC)

After 21 days of differentiation, organoids were collected for transcriptome analysis. Figure 4a–c shows principal component analysis (PCA) colored by experimental treatment, biological sex, and donor number. Herein, a robust transcriptional clustering of the three organoid types: basal (3 male, 2 female), fibrocartilaginous (4 female), and hypertrophic cartilage organoids (5 male, 1 female) was found. Within the hypertrophic donors, a potential subclustering could be distinguished, however as these subgroups are not apparent in other in vivo or in vitro measurements, it was not further investigated here. Following differential expression analysis, shown in Fig. 4d–e between the three groups, we found 4 496 differentially expressed genes (DEGs) between hypertrophic and undifferentiated organoids, but only 1 501 DEGs between fibrocartilaginous organoids and undifferentiated ones. Despite large histological differences, 1 356 genes showed a significant difference between hypertrophic and fibrocartilaginous callus organoids. A unique overlap approach is explained in Fig. 4f and allowed the identification of transcriptional markers that were specific to the organoid phenotypes. Gene ontology and pathway analysis confirmed that the 570 genes that are equal in the callus organoids, but different from the untreated controls, were related to skeletal system development, ECM organization, TGF-β signaling, and Hippo signaling pathways.

This included both chondrogenic markers such as SOX9, TGFβ3,^39^ COMP, SMAD6, CCN2,^40^ ID1-4 and FGFR3.^41^ Traditional chondrogenic markers ACAN and COL2A1 were also upregulated, but were significantly more expressed in hypertrophic organoids. Fig. S4 shows the expression of common chondrogenic and hypertrophic markers as determined by quantitative PCR. In addition, remodeling and mineralization markers such as ALPL,^42^ BMP2, MMP13,^43^ and SPP1 were also equally expressed. Interestingly, both fibrocartilaginous and hypertrophic organoids had a common expression of genes related to angiogenesis such as ANGPTL4 and PDGFA^44^ as well as small leucine-rich proteoglycan molecules such as BGN, LUM, and FMOD. Moreover, chondrogenic differentiation resulted in a decreased expression of oxidative stress genes, coagulation cascades, and glucocorticoid receptor pathways, all linked to inflammatory responses. All callus organoids also showed a decreased expression of progenitor genes such as GDF5,^45^ CLU^46^ and CTSK.^47^ Gene ontology analysis in Fig. 4k showed a common upregulation of chondrogenic genes and downregulation of progenitor genes, complement genes, and oxidative stress-related genes. Unique to the hypertrophic organoids were genes related to glucose metabolism such as ALDOC, PFKFB4 and SLC2A1, but also citrate deposition transporter gene SLC13A5.^48^ The hypertrophic identity was confirmed with specific expression of genes such as IHH,^49^ WNT4,^50^ IBSP,^51^ and LOXL2,^52^ with additional genes related to extracellular matrix stability such as COL11A1 and HAPLN1. Compared to both undifferentiated and fibrocartilaginous organoids, Fig. 4i–l and Fig. S5 showed that hypertrophic organoids showed a significantly decreased expression of another set of progenitor markers CXCL12,^53^ PTHLH,^54^ PDGFRB,^55^ and ADIPOQ.^54^ Fibrocartilaginous organoids, on the other hand, were characterized by an increase of elastic fibers such as ELN and FBLN5 and fibrillar collagens COL4, COL5, COL18.^56^ These showed a catabolic profile, with increased expression extracellular matrix-degrading enzymes MMP11,^57^ MMP19,^58^ PCSK5^59^ and SLC46A3.^60^ Moreover, they specifically expressed genes that inhibit TGF-β/BMP signaling such as IGF1,^61^ IGF2,^62^ GREM1 and GREM2^63^ and LRRC32.^64^ In Fig. S5, normalized bulk expression of known progenitor marker genes are shown. Undifferentiated organoids maintained a high expression of progenitor marker genes. However, a decreased expression of periosteal and other progenitor marker genes was observed in hypertrophic organoids. Fibrocartilaginous organoids also maintained a higher expression of these markers compared to undifferentiated organoids, and even a notable increase of osteoprogenitor markers, upregulation of PDPN, and downregulation of CD146.^65^

### Callus organoid secreted proteome analysis reveals specific biomarker candidates

**Fig. 5:**
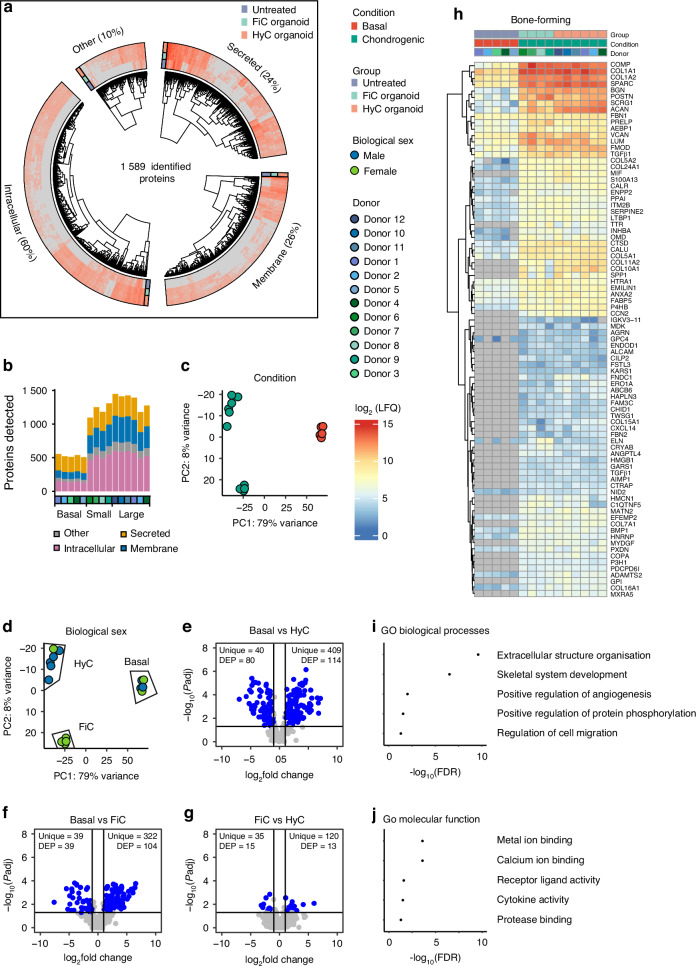
Secreted proteome identification. **a** Overview heatmap of robustly secreted proteins according to their cellular location. **b** Identified proteins per donor. **c**, **d** Principal component analysis. **e**–**g** Differential expression analysis of the identified secreted and membrane-associated proteins. **h** The panel of secreted proteins common to a successful cartilage-to-bone transition. **i** Gene ontology biological process enrichment of bone-forming proteins. **j** Gene ontology molecular function enrichment of bone-forming proteins

Bone forming callus organoids express common transcriptome signatures that are related to cell communication and signaling events. In order to elucidate the functional aspect, we collected conditioned medium from callus organoids following 21 days of differentiation and compared this to the conditioned medium of undifferentiated organoids. We used liquid chromatography with tandem mass spectrometry (LC-MS/MS) proteomics in combination with a data-independent neural network (dia-nn) for protein identification. In total, 2 421 proteins were identified throughout all samples. Following removal of low quantity reads and proteins with a coverage of less than 30% within the groups, 1 589 proteins remained. As shown in Fig. 5a, 60% of the identified proteins mapped to the intracellular space according to the UniProt database. 26% of the proteins were either membrane-spanning or membrane-associated and 24% were mapped to the extracellular space, being actively secreted. 10% of the identified proteins did not have a defined cellular localization. Figure 5b shows the total amount of proteins identified in each sample with at least 2/3 coverage in the technical replicates. The secretome of undifferentiated organoids contained on average 527 identified proteins, while an average of 1 558 proteins and 1 210 proteins were identified in the secretome of hypertrophic callus organoids and fibrocartilaginous callus organoids respectively. Principal component analysis, shown in Fig. 5c, d, reaffirmed the same clustering seen through morphological analysis (Fig. 3h), and transcriptome analysis (Fig. 4c), with a strong distinction between undifferentiated organoids (basal, *n* = 5), fibrocartilaginous callus organoids (FiC, *n* = 4 female) and hypertrophic cartilage callus organoids (HyC, *n* = 5 male, 1 female). Differentially expressed protein (DEP) analysis was performed, taking into account only the secreted and membrane-associated proteins (Fig. 5e–g) showing statistically significant differences between the secreted proteomes of organoid populations. Interestingly, this identified also a high number of robust exclusively secreted proteins within each group.

This analysis was extended to compare one group versus the rest (e.g., basal versus FiC and HyC), containing only proteins that are predicted to be actively secreted. As a result, four different protein panels were identified. Three panels, shown in Fig. S6, contain proteins specific and unique to either basal, fibrocartilage, or hypertrophic cartilage organoid secretomes. As such, hypertrophic callus organoids were marked by a secreted panel of 22 proteins, with 12 exclusive markers, including Integrin-binding Sialoprotein (IBSP),^51^ Chondroadherin (CHAD),^66^ Lysyl oxidase homolog 2 (LOXL2),^52^ Retinol-binding protein 4 (RBP4)^67^ and Angiopoietin-related growth factor (ANGPTL2).^68^ Fibrocartilaginous callus organoids secreted a unique panel of 5 proteins, including Sushi repeat-containing protein (SRPX2), an X-linked chondroitin sulfate proteoglycan found in growth plate cartilage,^69^ and shown to play a role in promoting migration.^70^ As shown in Fig. 5h, 84 proteins were specific to chondrogenic differentiation and equally present in the secretome of both hypertrophic and fibrocartilaginous callus organoids. 43 proteins, such as Periostin (POSTN),^71^ Aggrecan (ACAN),^72^ Tissue-nonspecific Alkaline Phosphatase (ALPL),^73^ and Annexin A2 (ANXA2),^74^ were also identified in the control group, but 41 proteins were exclusive to the treatment. These include Connective tissue growth factor (CCN2),^75,76^ Osteopontin (SPP1),^77^ Macrophage migration inhibitory factor (MIF),^78^ C-X-C motif chemokine 14 (CXCL14)^79^ and Agrin (AGRIN).^80^ Figure 5i–j shows enriched gene ontology terms for biological processes and molecular function of this common panel of 84 proteins. Together, they showed the proteins commonly secreted by FiC and HyC organoid populations were related to extracellular matrix modification through mineralization and protease activity as well as paracrine signaling towards cell migration, immune cell activation, and angiogenesis.

### Callus assembloid implants from both groups can form bone ossicles upon implantation

The commitment of an organoid towards a hypertrophic cartilage template or fibrocartilaginous template was decided within the first 7 days of chondrogenic treatment according to the data shown in Fig. 2. However, both callus organoids formed cartilaginous subtypes, with similar chondrogenic transcriptional profiles and a significant overlap in secreted protein panels, the question remains if this also affected their capacity to undergo ossification in vivo. Hence, we created donor-specific assembloids and implanted them subcutaneously in nude mice for 4 weeks.

**Fig. 6:**
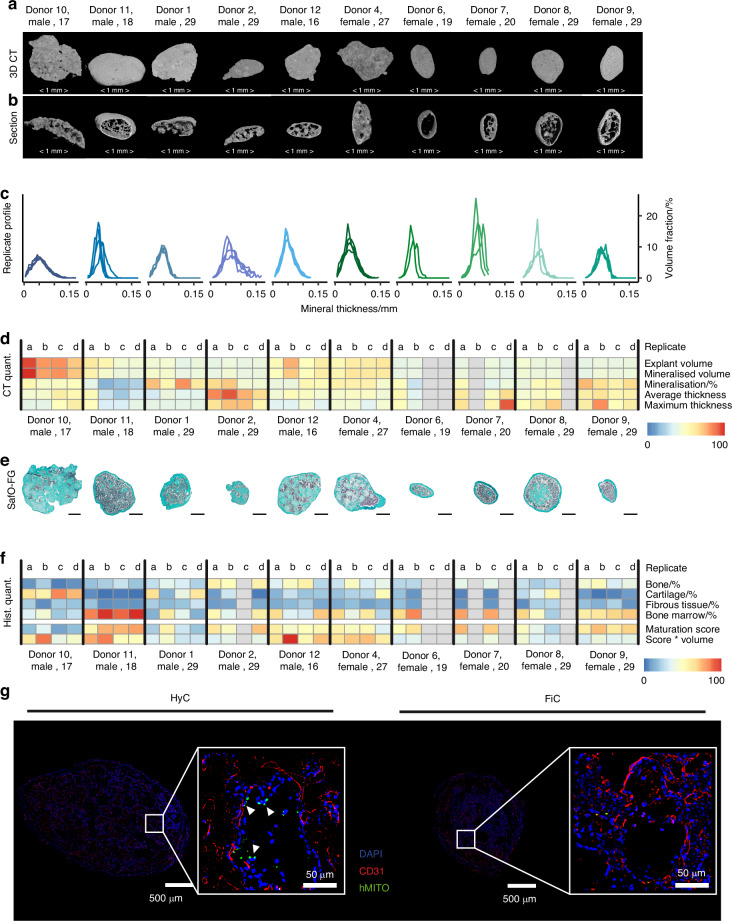
In vivo evaluation of donor-derived implants. **a** 3D microCT reconstruction of a representative explant per donor. **b** A cross-section of 3D microCT reconstructed explant. Scalebar = 1 mm. **c** Mineral thickness distribution profile of explant replicates, represented as standardized measures. **d** Quantification of 15ineralization from microCT. **e** Safranin-O/Fastgreen staining of a representative explant. Scale bar = 500 µm. **f** Histological quantification and explant scoring, represented as standardized measures. **g** Immunohistological staining of murine CD31 blood vessels and human mitochondria of representative explant slides from one hypertrophic organoid (HyC) and one fibrocartilage organoid (FiC) explant. Scalebars = 500 µm or 50 µm for the detail image

After 4 weeks of implantation, 85% of the implants were retrieved: 100% for HyC and 75% for FiC. With a minimum of 50% within each donor, both cartilage subtypes were capable of undergoing a cartilage-to-bone transition. In contrast, undifferentiated organoids in the negative control group did not form ectopic bone with a retrieval rate of 0%. Figure 6a, b shows representative 3D reconstructions of explants using microcomputed tomography (µCT). 3D reconstructions of all implants and their replicates per donor are shown in Fig. S7. After chondrogenic differentiation, all donor-specific implants were capable of mineralization in vivo with the formation of bone ossicles containing cortical bone, trabecular bone, and non-mineralized space within. As seen in Fig. 6c, the local mineral thickness distribution showed donor-specific profiles that were reproducible between replicates. However, µCT quantification in Fig. 6d showed a high biological variability in explant size, mineralized percentage, and mineralized thickness.

To further assess the extent in which a cartilage-to-bone transition is completed after 4 weeks in vivo, explants were stained for safranin O, fastgreen, and haematoxylin. This allowed the quantification of sulphated glycosaminoglycans, collagens in mineralized cartilage or bone tissue and embedded cells respectively. We used a machine learning pixel classification pipeline based on histological stain intensity and local variation as well as cell density, to segment bone, cartilage, bone marrow, and fibrous tissue. Representative sections are shown in Fig. 6e, with quantifications for all replicates in Fig. 6f. As a result, explants from all donor assembloids formed bone ossicles containing layered bone and bone marrow compartments. No fibrous tissue was found within the ossicles. The presence of mineralized cartilage varied between donors, indicating different stages of their cartilage-to-bone transition.

The ossicle maturation score was defined as a percentual calculation of bone and bone marrow area compared to cartilage and fibrous tissue area. This showed donor-dependent stages of the cartilage-to-bone transition, but no differences between fibrocartilaginous versus hypertrophic assembloids. However, since the same amount of starting cells were used for all implants, an additional overall score was defined taking into account both the ossicle maturation and produced volume. Here, implants from donors that followed the hypertrophic cartilage pathway showed an increased efficiency in producing a volume of high-quality bone tissue.

Finally, to better understand the potential mechanisms of action, the contribution of graft versus host was investigated. Figure 6g shows one explant for either hypertrophic organoid explants or fibrocartilage organoid explants, stained for human-specific mitochondria compared to murine CD31 as an endothelial marker. The extended dataset can be found in Fig. S8. We observed an extensive network of CD31-positive cells and blood vessels throughout all explants, especially in the bone marrow cavities. This was less dense in regions containing remaining cartilage, as well as the cortical and trabecular bone regions. In addition, while the majority of cells were derived from the murine host, the explants contained cells that stained positive for human mitochondria, especially in regions with cartilage tissues. However, we also found that explants from hypertrophic cartilage organoids contained more human cells lining the trabecular bone, but not the cortical bone. In explants from fibrocartilage organoids, the number of remaining human cells was lower, and they did not show a distinct localization pattern.

## Discussion

Advanced therapeutic medicinal products (ATMPs) are a novel class of therapeutics aimed at providing long-term solutions for unmet clinical needs.^81^ Tissue engineered ATMPs are complex three-dimensional living products and the translation of these novel products to clinical practice and marketing authorization is hampered by a lack of quality attributes able to predict implant functionality.^82,83^ In addition the fact that a substantial number of such products are at present still autologous adds the challenge of deciphering and mapping the impact of donor variability on product CQAs. Even for allogenic products, batch-to-batch variability remains a challenge. The identification of donor-independent, non-invasive biomarkers able to define release criteria for ATMPs is a prerequisite to enable subsequent translation to the clinic.^84,85^ One promising strategy for the repair of bone defects that is having an increased impact on the field of skeletal tissue engineering, is to mimic the fracture callus. Here, the generation of provisional cartilaginous implants that recapitulate robust fracture healing events results in a close-to-native bone regeneration. Such tissue-engineered cartilage-intermediates have been developed using diverse progenitor cell types, including bone marrow,^86,87^ adipose-derived cells,^88,89^ nasal chondrocytes,^90^ umbilical cord blood-derived cells,^91^ induced pluripotent stem cells.^92^ However recently the use of periosteum-derived progenitor cells^86,93–96^ is more and more being explored due to the fact that this cell type is the predominant contributing cell in fracture callus formation and bicortical fracture repair.^16^ We have recently reported on the formation of periosteum-derived callus organoids which were able to bridge critical-size tibial defects.^24,97^ In the current work, we show exceptional functional robustness of this TE strategy across biological variation. However, there is still lack of solid information regarding the influence of donor-to-donor variability on the process of endochondral ossification of tissue-engineered implants. In this work, we seek to address this gap by defining donor-independent secreted biomarker panels reflective of the implants’ bone-forming capacity.

We discovered two distinct engineered callus organoid groups exhibiting diverse in vitro quality properties which we termed as: “(pre)hypertrophic” callus organoids (HyC) with pronounced cartilaginous ECM compartment and phenotypical chondrocytes, and smaller “fibrocartilaginous” callus organoids (FiC) which did not exhibit these characteristics. Our results indicate that engineered hypertrophic cartilage was not a prerequisite of endochondral bone formation. Still, this pathway is more efficient in creating tissue volume and we found indications of a higher cellular contribution of the hypertrophic cells in the remodeled tissue. In fact, it is now accepted that hypertrophic chondrocytes are prone to cell plasticity leading to transdifferentiation of chondrocytes into osteoblasts, adipocytes, and new progenitor reservoirs.^53,98^ The bone-forming potency of human fibrocartilage organoids is an important finding as it might be further explained by recently published work on endochondral bone formation in the context of murine fracture repair which supports a main role for its fibrocartilaginous component.^99^ Moreover, single-nuclei trajectory analysis performed by Perrin et al. identified periosteum-derived injury-induced POSTN and ASPN-expressing fibrogenic cells as drivers of murine bone regeneration through paracrine signaling.^100^ Both proteins were also identified in the current study in the secretome of human bone-forming callus organoids. Interestingly, these proteins might be part of a larger panel orchestrating cell migration, angiogenesis, and immune cell modulation. Cartilage-to-bone transition is driven by the balance of matrix-producing osteoblasts and matrix-degrading chondroclasts and osteoclasts, both of which are regulated by different cells of the immune system.^17^ This balance determines the remodeling kinetics and the final bone quality.^101^ Hence, we hypothesize potential long-term differences in bone quality depending on the ratio of immune-modulatory signaling proteins secreted by cartilaginous implants.

Another intriguing observation is that periosteal cells from all male donors generated hypertrophic organoids, while cells from 4 out of 5 female donors generated fibrocartilage organoids, indicating a role for biological sex in the mechanism of periosteal bone repair. The role of biological sex and hormone regulation is one of the known factors affecting bone homeostasis in age and diseases,^19,102^ as well as cartilage health.^103^ For fracture repair, sexual dimorphism was recently investigated in murine models, showing that male mice display more robust fracture healing, with larger callus sizes containing higher cartilage tissue fraction in male mice compared to female mice.^26,104^ Then, a sex-dimorphism was described in two-dimensional culture of human femoral head skeletal stem cells (SSCs), showing similar differentiation capacity but differences in proliferation and cell size.^105^ Moreover, a study by Andrew et al. showed an impaired fracture repair in female ovariectomized mice, which was rescued by administration of systemic estrogen hormone therapy. This was similarly confirmed in vitro for 2D culture of human femoral head SSCs, showing biological sex-specific activation and proliferation of progenitor cells.^106^ Our data confirm this sexual dimorphism in a 3D human organoid model, with a more distinct proliferation and extensive cartilage volume production in male versus female organoids. Still, all organoids showed a decreased expression of periosteal progenitor genes^107^ signifying that during the differentiation process, progenitors actively committed to the chondrogenic or osteogenic lineage. When comparing our data to the most recent periosteal stem and progenitor marker genes, reviewed by Trompet et al.,^108^ distinct sets of progenitor genes, displayed in Fig. S5, were differentially expressed in our data potentially driving different routes towards bone formation. This also shows differences in marker genes related to endosteum-derived progenitor cells, which may point towards a larger heterogeneity or plasticity in the periosteal progenitor population.

Limitations of this study include the use of ectopic bone formation in athymic immune compromised female mice, which gives insufficient insight in the role of the immune system and potentially leads to different remodeling kinetics between male and female organoid due to the presence of a female systemic environment.^106,109^ Finally, the heterogeneity of starting cell populations, or final implant cellular content was not investigated, as it lies outside the scope of this study. However, this might be a promising subject of future studies towards cell selection procedures enabling more generalized differentiation protocols downstream. Here, the effect of hormones and animal models for both biological sexes will be explored in future studies. Quantitative validation of the identified secreted protein panels throughout the differentiation protocol is needed to define predictive critical quality attributes in a broader context. This includes a broader biological variability, a validation as autologous implants in immune-competent large animal models as well as the interaction to the final manufacturing protocol to be used in a (pre)clinical setting.

In conclusion, engineered human periosteum-derived callus organoids, are promising building blocks for the bottom-up biofabrication of TE-ATMPs for the healing of bone defects. We show this organoid model is a close representation of the periosteal contribution to the human fracture callus and marked by the ability to identify biological phenomena such as the role of biological sex, the fibrogenic component of the fracture callus, and potential heterogeneity in periosteal progenitor populations leading to distinct callus phenotypes and cartilage-to-bone transition pathways. Moreover, we show functional robustness across biological variation, linked to specific panels of secreted proteins providing a promising source of measurable biomarker candidates to be further investigated as tools for quality monitoring and potency prediction.^110–112^ These biomarkers could be non-destructively assessed as critical quality attributes and could act as a quality target profile indicating time points where skeletal tissue-engineered products would be ready for implantation, to reduce cost, minimize failures, and enhance clinical translation.^113^ Still, the observed heterogeneity may have a significant impact towards clinical translation, potentially requiring different protocols specific for patient populations based on biological sex,^114^ including the addition of hormones at the proliferation stage,^106,115,116^ or adaptive supplementation of additional growth factors to guide cell fate towards the hypertrophic lineage.^117^ While we identified a panel of donor-independent biomarker candidates, different protocols might also require their own quality markers and even manufacturing strategy. Nevertheless, a key finding was that despite the pathway of cartilage–to–bone transition the formation of high-quality ossicles was achieved indicating the robustness these tissue-engineered organoid building blocks.

## Materials and methods

### Experimental design

The objective of the study was to obtain a better understanding of biological variability and how biological sex affects the bone-forming capacity of tissue-engineered transient cartilage implants. Implants were generated using the protocol described in Fig. 1a, where human periosteum-derived cells are collected, expanded for 7 passages, and cultured as organoids for 21 days. Bone-forming capacity is evaluated using the ectopic mouse model. We used cells collected from 12 human donors. The experimental design is described in Fig. 1f, where 3 experimental groups are created during the organoid phase: (1) not stimulated, *n* = 5, (2) male, chondrogenic differentiation, *n* = 5, (3) female, chondrogenic differentiation, *n* = 5. To elucidate the requirements for bone formation, we combined histological, transcriptome, and secretome analysis of organoids and their conditioned medium at day 21, right before in vivo implantation.

### hPDC harvest and expansion

Human periosteum-derived cells (hPDCs) were isolated from periosteal biopsies of 12 different donors (6 male, 6 female, 16–29) as previously described.^118^ The hPDCs were expanded until passage 7 (13.7 ± 3.4 cumulative population doublings) at 5 700 cells/cm^2^, 37 °C, 5% CO_2,_ and 95% humidity in Dulbecco’s modified Eagle medium (DMEM, Gibco, UK) with 10% fetal bovine serum (South Afrika FBS, BioWest, France) and 1% antibiotic-antimycotic solution (Invitrogen, USA). The medium was changed every 3–4 days, and cells were harvested with TrypLE Express (Life Technologies, UK) at a confluence of 80%. The ethical committee for Human Medical Research (Katholieke Universiteit Leuven) approved all procedures, and patients’ informed consent forms were obtained (ML7861).

### Callus organoids

The commercially available microwell platform (AggreWell™800, STEMCELL Technologies Inc, Canada) was coated with Anti-Adherence Rinsing Solution (STEMCELL Technologies Inc, Canada) to avoid cell attachment, centrifuged to ensure homogenous coating, and washed with LG-DMEM (Gibco) supplemented with 1% antibiotic– antimycotic (Invitrogen) before cell seeding. hPDCs were harvested with TrypLE Express (Life Technologies, UK) and seeded at 300 000 cells per well to contain 1 000 cells per microwell upon sedimentation. The undifferentiated cells were cultured in a serum-free lipid-free, low carrier protein basal medium containing LG-DMEM (Gibco) supplemented with 1% antibiotic– antimycotic (Invitrogen), 1 × 10^−3 ^mol/L ascorbate-2 phosphate, 1 × 10^−7 ^mol/L dexamethasone, 40 μg/mL L-proline, 20 × 10^−7^ mol/L of Rho- kinase inhibitor Y27632 (Axon Medchem), ITS Premix Universal Culture Supplement (containing 6.25 μg/mL insulin, 6.25 μg/mL transferrin and 6.25 ng/mL selenious acid, Corning). For chondrogenic differentiation, this was additionally supplemented with 100 ng/mL BMP2 (INDUCTOS), 100 ng/mL GDF5 (PeproTech), 10 ng/mL TGF-β1 (PeproTech), 1 ng/mL BMP-6 (PeproTech), and 0.2 ng/mL basic FGF-2 (R&D systems). Differentiation medium was prepared in advance in batches for multiple experiments, aliquoted, and stored at −20 °C until use. Half of the medium was changed on days 3, 7, 10, 14 and 17.

### Assembloid formation and implantation

Custom round-bottom macrowells were created in 3% agarose (w/v) (Invitrogen) and sterilized using UV. Microtissues were gently flushed out from their microwells on day 21, concentrated, and added to the macrowells at 600 microtissues per well to sediment for 1 h. CM was added and the constructs were incubated for 24 h at 37 °C, 5% CO_2_, and 95% humidity to fuse into a coherent implant. Implants were removed from the agarose macro well, washed in 1xDPBS to remove loosely bound growth factors, and implanted subcutaneously in the back at the shoulder region of immune-compromised female mice between 8–20 weeks old (*Rj*:NMRI^nu/nu^). After 4 weeks, the implants were taken out and fixed for 4 h in 4% PFA. All procedures on animal experiments were approved by the local ethical committee for Animal Research, KU Leuven. The animals were housed according to the regulations of the Animalium Leuven (KU Leuven).

### Microcomputed tomography (µCT)

3D quantification of mineralized tissue in PFA-fixed explants was done through micro-CT (Pheonix Nanotom M, GE Measurement, and Control Solutions). Explants were scanned with a diamond target, mode 0, 500 ms exposure time, 1 frame average, 0 image skip, 2 400 images, and a 0.1 mm aluminum filter. Samples were scanned with 60 kV and 140 µA. CTAn (Bruker micro-CT, BE) was used for all image processing and quantification of mineralized tissue based on automatic Otsu segmentation, 3D space closing, and despeckle algorithm. The percentage of mineralized tissue was calculated with respect to the total explant volume. CTvox (Bruker micro-CT, BE) was used to create 3D visualization.

### Sampling and storage

300 pooled microtissues (*n* = 3/4) were gently flushed out from their microwells at different time points (*t* = 5 h, day 7, day 14, day 21) and centrifuged (10 min, 1 300 r/min) to separate microtissues from conditioned medium. Conditioned medium was aliquoted, frozen in liquid nitrogen, and stored at −80 °C. Microtissues were lysed in 350 µL RLT lysis buffer (Qiagen, Germany) supplemented with 3.5 µL β-mercaptoethanol (Sigma Aldrich, Germany), vortexed and stored at −80 °C.

### DNA quantification, RNA isolation

DNA assay kit QuantiT dsDNA HS kit (Invitrogen) was used to quantify the DNA content from cell lysate according to the manufacturer’s protocol. RNA was isolated from the lysate with the RNeasy Mini Kit (Qiagen) according to the manufacturer’s protocol and quantified with NanoDrop 2000 (Thermo Scientific). RevertAid H Minus First Strand cDNA Synthesis Kit (Thermo Scientific) was used for reverse transcription. 1 µg oligo^(dT18)^ was added to 11 µL RNA for 5 min at 65 °C, the reaction mixture (4 µL 5× reaction buffer, 1 µL ribolock ribonuclease inhibitor, 2 µL dNTP mix (10 × 10^−3^ m), and 1 µL RevertAid H Minus M-MuL VRT) was added, cDNA was generated using the Applied Biosystems Veriti 96-Well Fast Thermal Cycler (60 min at 42 °C followed by 10 min at 70 °C) and diluted in RNase-free water to 5 ng/mL.

### Transcriptome analysis

The Genomics Core Leuven performed the sequencing as follows. Library preparation was performed with the Lexogen QuantSeq FWD Sample Preparation Kit, according to the manufacturer’s protocol. Libraries were sequenced on the Illumina HiSeq4000 sequencing system. Protocol Quality control of raw reads was performed with FastQC v0.11.7.^119^ Adapters were filtered with Trimmomatic v0.39.^120^ Splice-aware alignment was performed with STAR^121^ against the reference genome using the default parameters. Reads mapping to multiple loci in the reference genome were discarded. Quantification of reads per gene was performed with FeatureCounts from Subread package.^122^ Count-based differential expression analysis was done with R-based (The R Foundation for Statistical Computing, Vienna, Austria) Bioconductor package DESeq2.^123^ Reported *p*-values were adjusted for multiple testing with the Benjamini-Hochberg procedure, which controls false discovery rate (FDR).

### Histology

600 pooled microtissues were gently flushed out from their microwells, concentrated, and fixed in 2% PFA overnight, mixed in 3% agarose, dehydrated, and embedded in paraffin overnight. Ectopic explants were fixed in 4% PFA for 4 h, decalcified in ethylenediaminetetraacetic acid (EDTA)/PBS (pH 7.5) for 10 solution changes at 4 °C, dehydrated, embedded in paraffin overnight, and sectioned at 5 µm thickness. For safraninO (Sigma) staining, sections were deparaffinized and rehydrated, counterstained with hematoxylin (Merck, cat 6525) for 1 min, briefly dipped in acid alcohol (1% HCL in 70% EtOH), rinsed in water, stained with 0.03% Fast Green (KLINIPATH, cat 80051) and then dipped in 1% glacial acetic acid followed by a 7 min staining in 0.25% safraninO (KLINIPATH, cat 640780). For alcian blue staining, slides were deparaffinized, rehydrated, and stained for 30 min with freshly filtered alcian blue (Alcian blue 8GX, Sigma). After a washing cycle, the slides were counterstained for 5 min with aqueous nuclear fast red (lab vision, ref TA-060-NF). Then the samples were washed in tap water, dehydrated with an ethanol series, cleared in Histoclear, and mounted in Pertex for microscopy imaging.

### Immunohistochemistry

Microtissues were fixed in 4% PFA at 4 °C for 1 h, embedded in paraffin, and sectioned into 5-µm thick slices. The slices were deparaffinized twice in 100% HistoClear for 5 min and rehydrated in the following decreasing concentration gradient of ethanol: 96%, 70%, and 50%. Antigen retrieval was performed by boiling in Tris-EDTA buffer (10 mmol/L Tris base, 1 mmol/L EDTA solution, pH 9.0) for 30 min for OSX and IHH staining, or by enzymatic treatment with 0.1 mg/mL pepsin in 0.2 mol/L HCl for 15 min at room temperature. Nonspecific reactivity was minimized by incubation in blocking buffer (PBS with 2.5% BSA) at room temperature for 30 min. Primary antibodies were diluted in PBS with 2.5% BSA and 0.3% triton-X100. Tissue sections were incubated overnight at 4 °C with the following concentrations and combinations: 1:50 mouse anti-human Ihh Antibody (H-12) (sc-271101, Santa-Cruz), 1:200 mouse anti-human COL1A Antibody (COL-1) (sc-59772, Santa-Cruz) with 1:50 rabbit anti-human collagen type II polyclonal antibodies (AB761, Merck). After washing three times 10 min in PBS, tissue slices were incubated with secondary antibodies overnight at 4 °C: 1:500 Goat anti-Mouse IgG (H + L), Alexa Fluor™ 488 (A-11001, Invitrogen), or a combination of 1:500 Goat anti-Mouse IgG (H + L), Alexa Fluor™ 488 and 1:500 Donkey anti-Rabbit IgG (H + L) Highly Cross-Adsorbed Secondary Antibody, Alexa Fluor™ Plus 594 (A-32754, Invitrogen) in the same buffer as primary antibodies. Nuclear counterstain was performed using 1:2 000 SYTOX Deep Red Nucleic Acid Stain (S11380, Invitrogen) for 30 min at room temperature. Slides were mounted in Mowiol 4–88 (Sigma-Aldrich; Merck KGaA).

### Confocal and 2-photon imaging

Confocal and two-photon z-stacks were recorded on an LSM 780 microscope (Zeiss) using a 25x, water-immersion objective (NA 0.8, Zeiss). For confocal z-stacks Alexa-594, SYTOX Deep Red Nucleic Acid Stain were excited by 561 or 633 respectively. Emission signals were descanned and collected with spectral detectors. A tuneable Mai Tai DeepSee Titanium-Sapphire femtosecond laser (680–1 050 nm; Spectra-Physics) was used for recording 2-photon z-stacks of forward second harmonic generation, Alexa-488 with an excitation wavelength set at 850 nm. Images were corrected for brightness and contrast using Fiji.

### Histochemistry quantification of organoids and explants

Alcian blue quantification of organoid extracellular matrix (ECM). For each donor, 3 organoids were stained with alcian blue and imaged with an Olympus IX83 inverted microscope equipped with a DP73 camera. Fiji (ImageJ) deconvolution2^124^ macro was used to isolate alcian blue intensity, leading to an average intensity measure within the region of interest. SafO explant quantification. For each explant, 3 slides were stained with SafO/FastGreen and imaged with an Olympus IX83 inverted microscope equipped with a DP73 camera. A region of interest was selected in ImageJ, and imported in Ilastik for pixel classification.^125^ Five tissue types were distinguished as shown in Fig. S8 Pixel prediction images were exported to ImageJ and each tissue type was quantified as a percentage relative to the total amount of pixels in the region of interest.

### Secretome analysis

#### Sample preparation

Proteins contained in culture supernatants were precipitated via 25% v/v TCA precipitation (4 °C; 20 min). Precipitated proteins were pelleted via centrifugation (20 000 × *g*; 20 min; 4 °C), on a bench-top centrifuge. The pellet was washed twice with ice-cold acetone and re-pelleted via centrifugation (20 000 × *g*; 20 min; 4 °C). The protein pellet was then solubilized in 8 mol/L Urea in 1 mol/L ammonium bicarbonate solution (ABS). Polypeptide concentrations were measured using the Bradford reagent. Polypeptides were separated by 12% SDS-PAGE and visualized by silver staining.^126^

#### LC-MS/MS: Sample preparation, acquisition, and DIA-NN data analysis

Proteins contained in culture supernatants were precipitated via 25% v/v TCA precipitation (4 °C; 20 min). Precipitated proteins were pelleted via centrifugation (20 000 × *g*; 20 min; 4 °C), on a bench-top centrifuge. The pellet was washed twice with ice-cold acetone and re-pelleted via centrifugation (20 000 × *g*; 20 min; 4 °C). The protein pellet was then solubilized in 8 mol/L Urea in 1 mol/L ammonium bicarbonate solution (ABS). Polypeptide concentrations were measured using the Bradford reagent. Polypeptides were separated by 12% SDS-PAGE and visualized by silver staining.^126^

Tryptic peptides were resuspended in 20 μL of H_2_O containing 0.1% formic acid (FA). Approximately 10 μL (around 500 nanograms) were loaded onto an Evotip and subjected to analysis using an Evosep One LC system (EVOSEP), coupled to a ZenoTOF 7600 mass spectrometer equipped with an Optiflow source (SCIEX). The method employed on the Evosep One was the 30SPD (samples per day; 44 min gradient) with the EV1137 Performance column (15 cm × 150 µm, 1.5 µm). Operating in positive mode, the mass spectrometer utilized a OptiFlow V source, spray voltage of 4 500 V, and employed SWATH mode. This SWATH acquisition scheme comprised 85 variable-size windows, covering a precursor mass range of 350–1 250 m/z, with an accumulation time of 0.02 s.

Raw data was processed using DIA-NN 1.8.1 (Data-Independent Acquisition by Neural Networks) (PMID: 31768060) in library-free mode. These spectra were compared against the Human reference proteome database (https://www.uniprot.org/proteomes/UP000005640). Enzyme specificity was defined as C-terminal to arginine and lysine, allowing cleavage at proline bonds with a maximum of one missed cleavage. Variable modifications included oxidation of methionine residues and N-terminus acetylation, while the fixed modification was carbamidomethylation of cysteine residues. Most of DIA-NN’s default settings were retained, utilizing ‘robust LC (high precision)’ mode with retention time (RT)-dependent normalization enabled, except for enabling the match between runs (MBR) option.

Using custom scripts in R, low reads (<5) were removed as well as all proteins with an identification coverage below 30% within the experimental groups and without at least 70% coverage in one of the conditions. Differential expression analysis was performed using log_2_(n + 1) normalized reads, protein-wise *t*-test, and Benjamin-Hochberg correction for multiple testing. Uniprot cellular localization with manual correction was used to retain actively secreted and membrane-associated proteins. Conditions for specific biomarker panels for an experimental group were a sample coverage of 100%, log_2_ fold change (Log_2_FC) > 2, and False Discovery rate (FDR) < 0.05. For the treatment-specific marker panel, proteins were retained with a sample coverage of 100% for chondrogenically treated organoids, (Log_2_FC) > 2, and False Discovery rate (FDR) < 0.05 compared to the undifferentiated control. Here, an additional criterion was added that no differences exist between hypertrophic and fibrocartilaginous callus organoids, with a Log_2_FC < 2 and FDR > 0.05.

### Statistical analysis

All statistical analyses were performed using standard functions in R (R core team). Statistical significance was defined at *P* < 0.05. Comparing two means was done through a two-sided, unpaired *t*-test. For non-normal data, a Wilcoxon test was performed. For multiple comparisons, an ANOVA analysis followed by Tukey’s post hoc test was used. Data is presented as mean and standard deviation from four samples. Symbols used are *P < 0.05, **P < 0.01, ***P < 0.001, and ****P < 0. 000 1.

## Supplementary information

20240807_supplement_boneres.docx

## Data Availability

The data that support the findings of this study are available from the corresponding author [IP], upon reasonable request. The raw and processed RNA sequencing data are deposited at the Gene Expression Omnibus under accession code GSE271551.
