# Supplementary material for: Callus organoids reveal distinct cartilage to bone transition mechanisms across donors and a role for biological sex
Source: Bone Res. 2025 Mar 26;13:41. doi: 10.1038/s41413-025-00418-z (PMC11947321; doi:10.1038/s41413-025-00418-z)
Supplement: Supplementary file 1 — 20240807_supplement_boneres.docx [file 41413_2025_418_MOESM1_ESM.docx]

Supplementary Materials for

**Callus organoids reveal distinct cartilage to bone transition mechanisms across donors and a role for biological sex**

Isaak Decoene^1,2^, Hanna Svitina^1,2^, Mohamed Belal Hamed^4^, Anastassios Economou^4^, Steve Stegen^2,5^, Frank P. Luyten^1,2^, Ioannis Papantoniou^1,2,3*^

*Corresponding author. Email: [ioannis.papantoniou@kuleuven.be](mailto:ioannis.papantoniou@kuleuven.be)

**This PDF file includes:**

Figs. S1 to S9


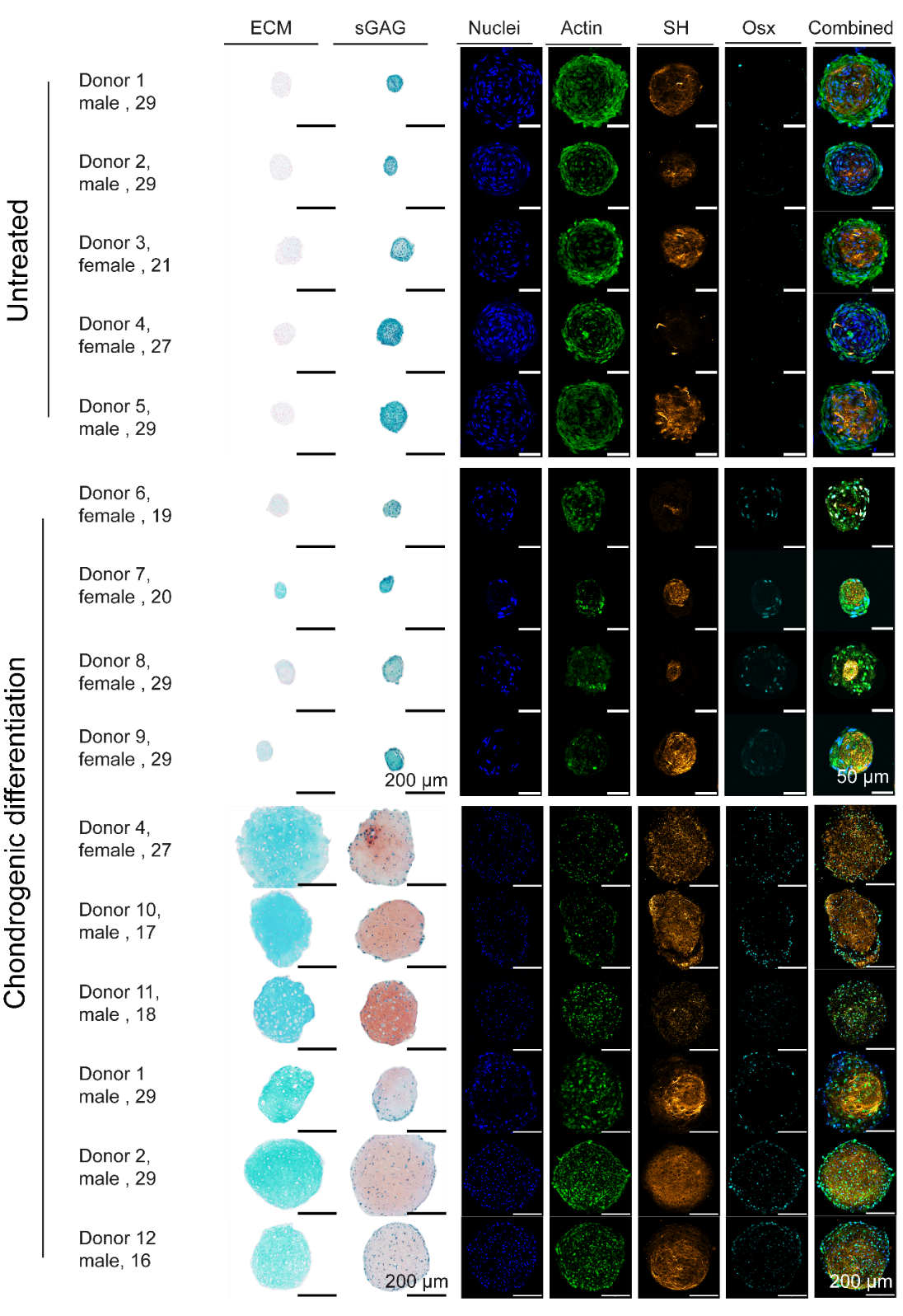


Fig. S1.

Extended dataset showing one representative organoid per donor, stained for cartilage extracellular matrix (ECM), suphated glycosaminoglycans (sGAG), nuclei, actin, structured extracellular matrix fibers through second harmonic (SH) generation imaging, and osteogenic marker Osterix (OSX). Scalebars are indicated on the figure.


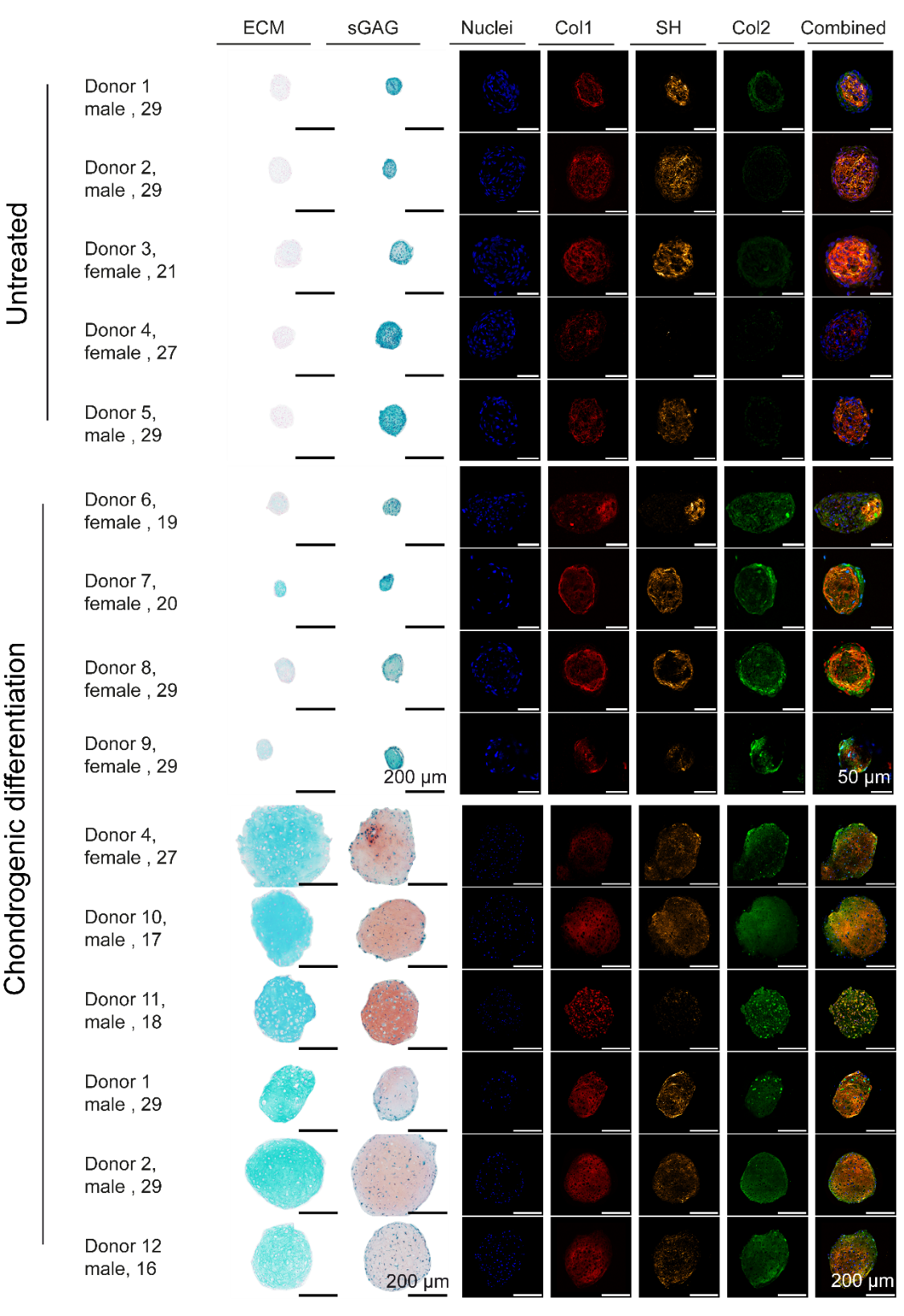


Fig. S2.

Extended dataset showing one representative organoid per donor, stained for cartilage extracellular matrix (ECM), suphated glycosaminoglycans (sGAG), nuclei, Collagen I (COL I), structured extracellular matrix fibers through second harmonic (SH) generation imaging, and chondrogenic marker Collagen II (COL II). Scalebars are indicated on the figure.


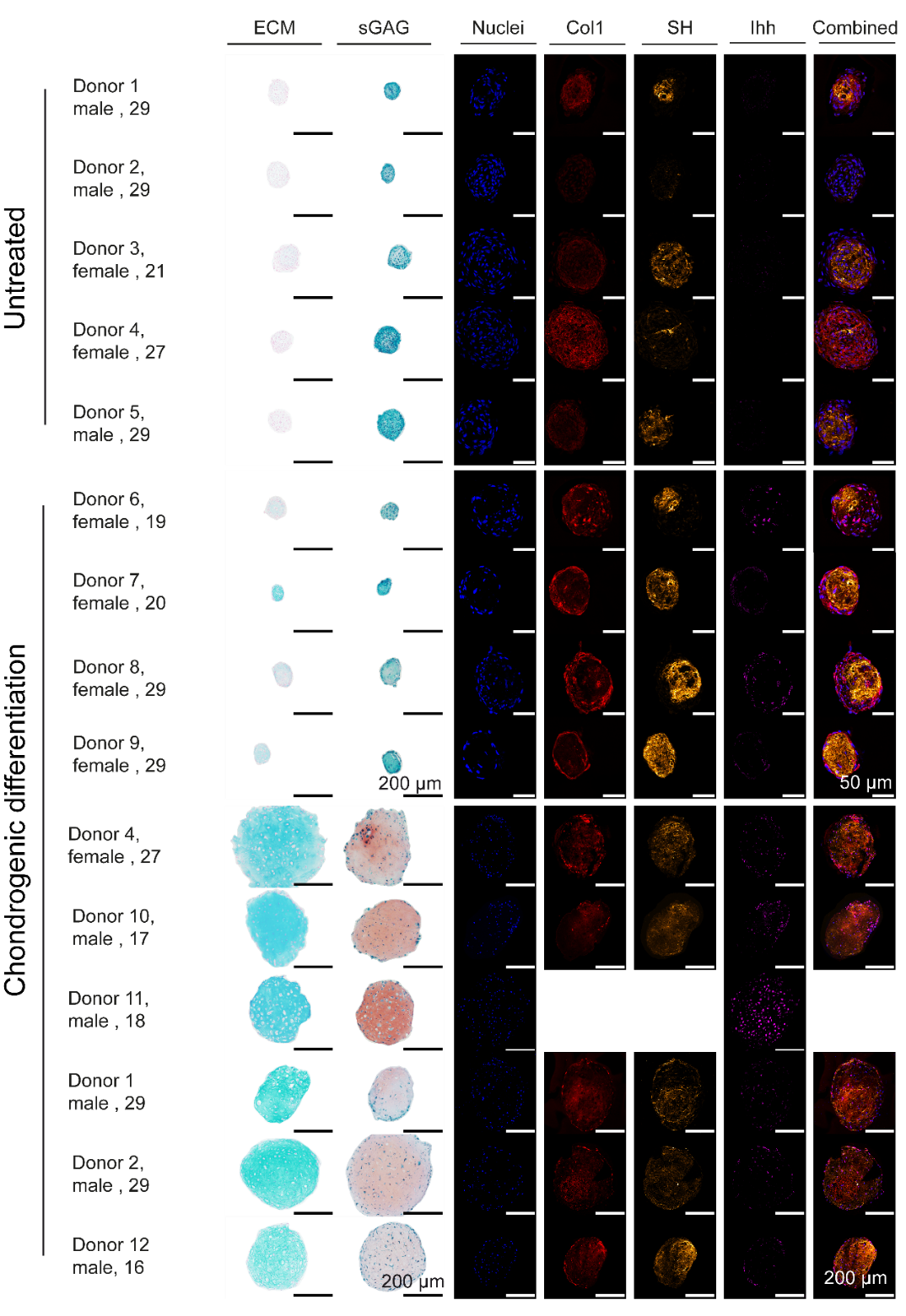


Fig. S3.

Extended dataset showing one representative organoid per donor, stained for cartilage extracellular matrix (ECM), suphated glycosaminoglycans (sGAG), nuclei, Collagen I (COL I), structured extracellular matrix fibers through second harmonic (SH) generation imaging, and prehypertrophic marker Indian Hedgehog (IHH). Scalebars are indicated on the figure.


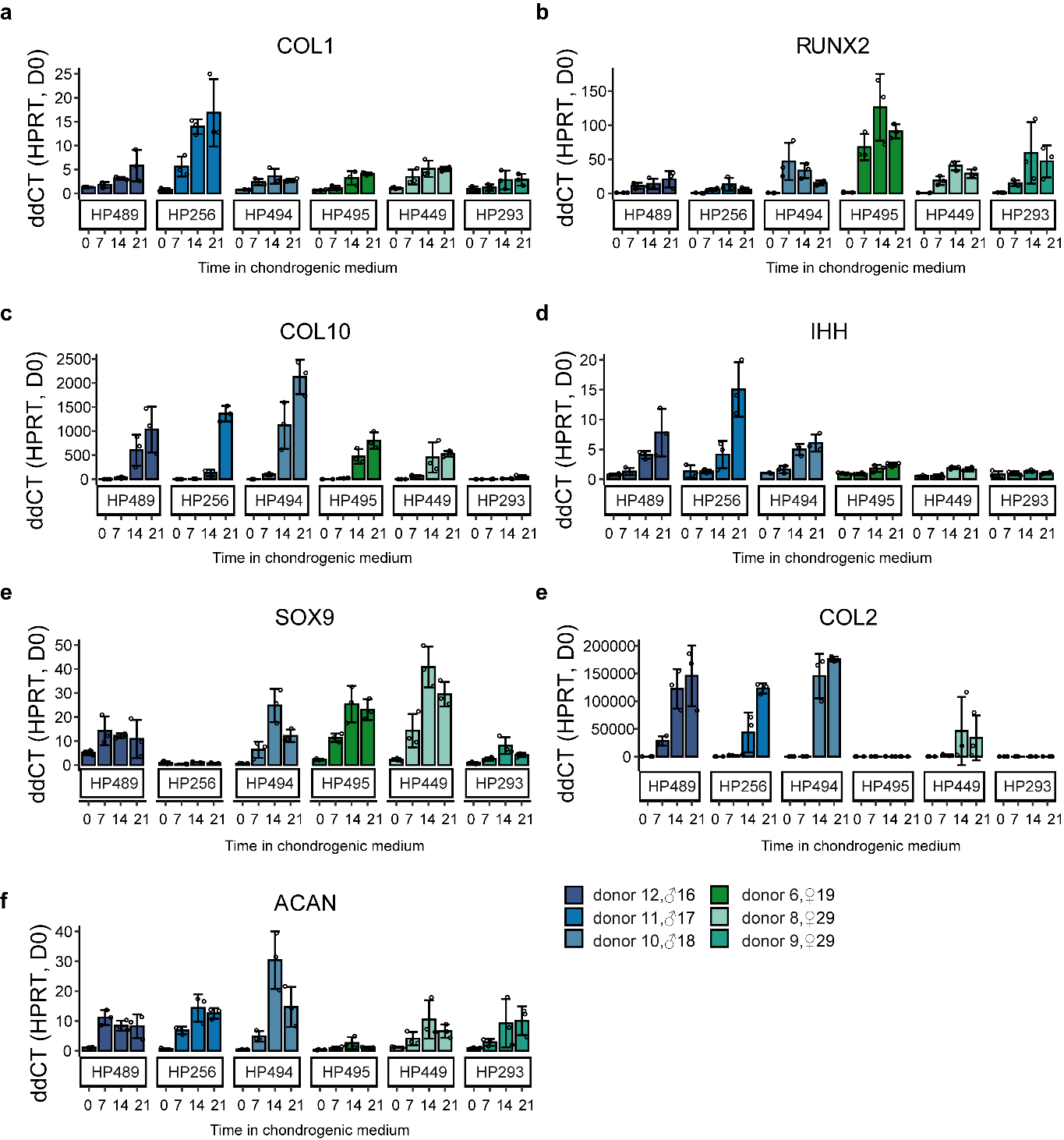


Fig. S4.

Quantitative PCR over time, displayed per donor. (a) COL 1, (b) RUNX2, (c) COL 10, (d) IHH, (e) SOX9, (f) COL 2, (g) ACAN. Data is represented per donor as mean +-standard deviation (n=3).


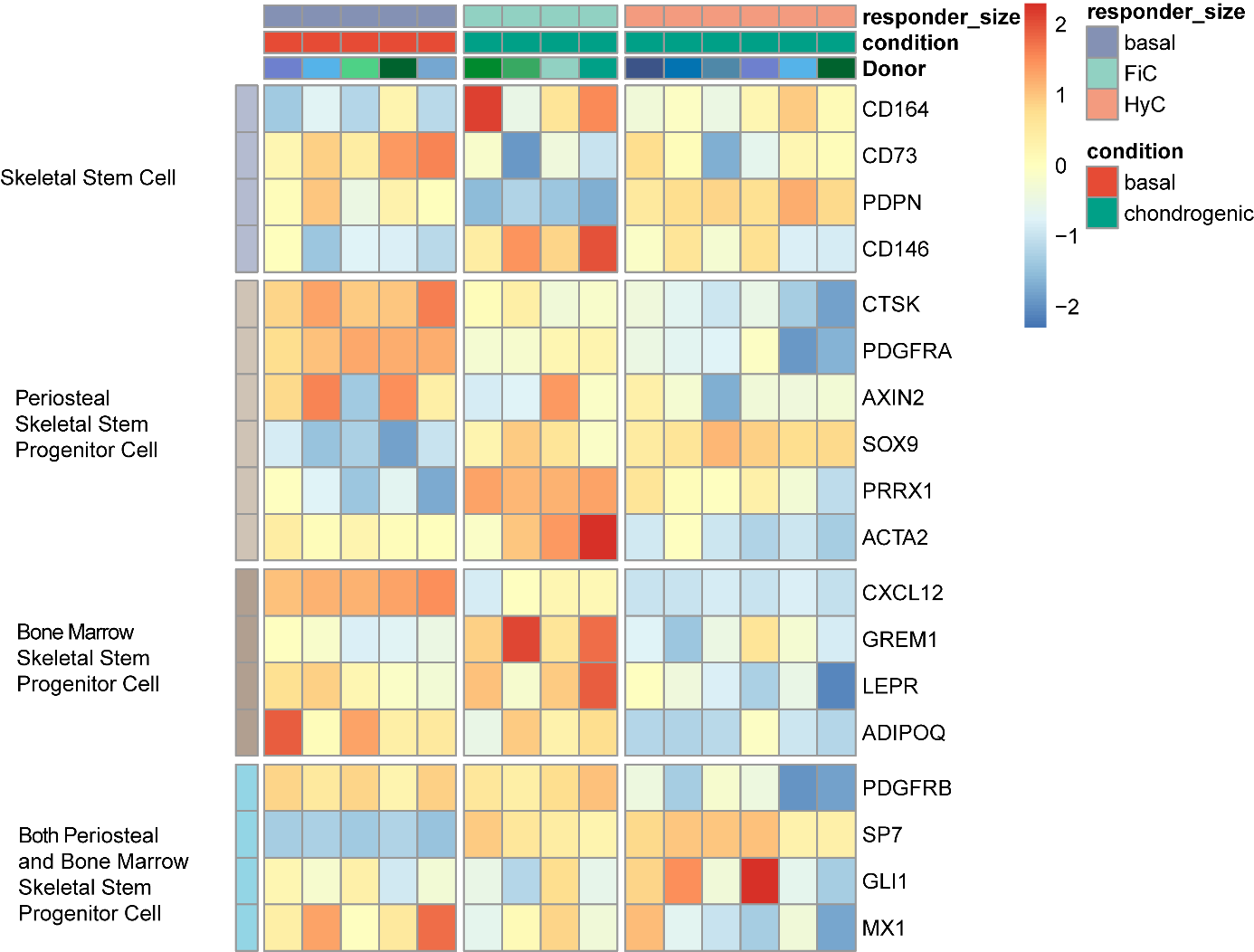
Fig. S5.

Summary of progenitor marker gene expression profiles determined from transcriptome analysis grouped by Skeletal stem cell markers ^58^, and periosteal versus bone marrow skeletal stem progenitor cells ^74^. Data represented as gene-wise standardized expression.


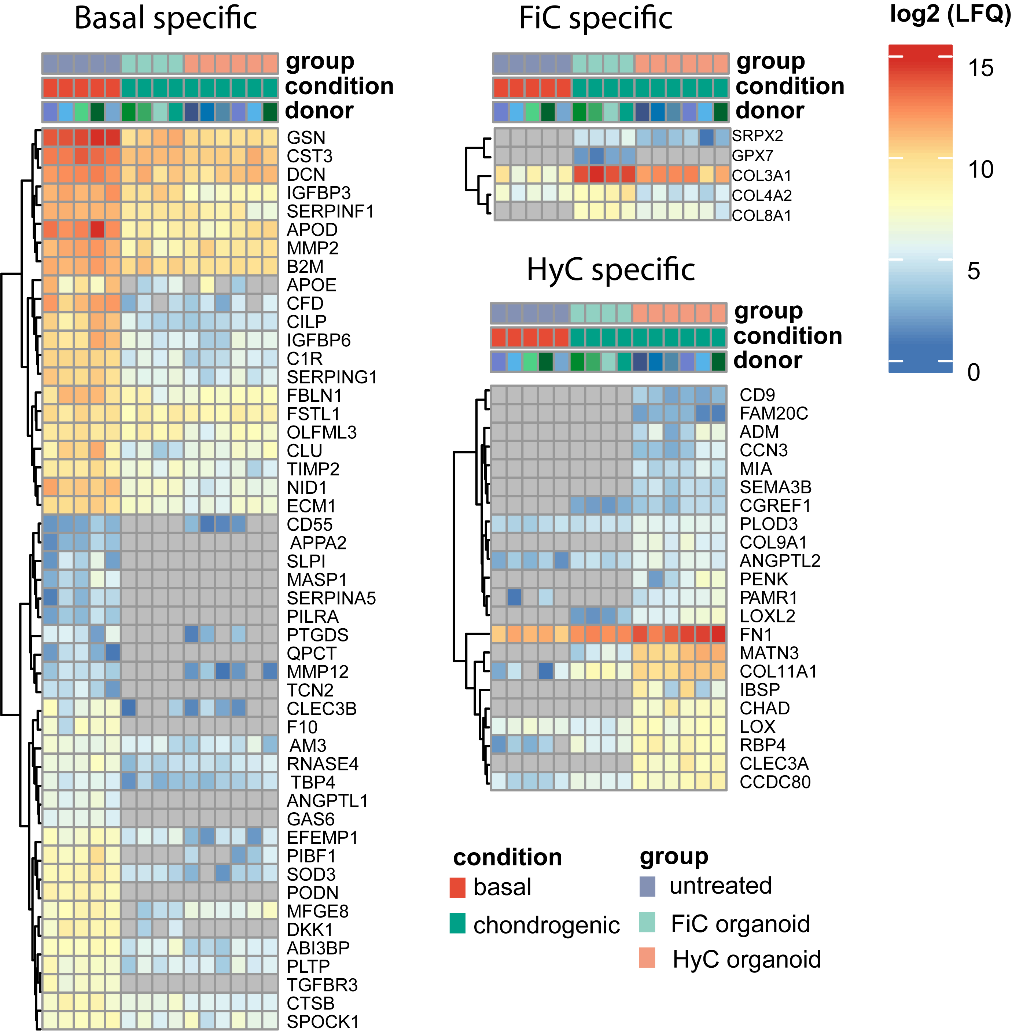


Fig. S6.

Secreted protein panel specific for basal, fibrocartilage (FiC) and hypertrophic cartilage (HyC) organoids. Data represented as normalized expression.


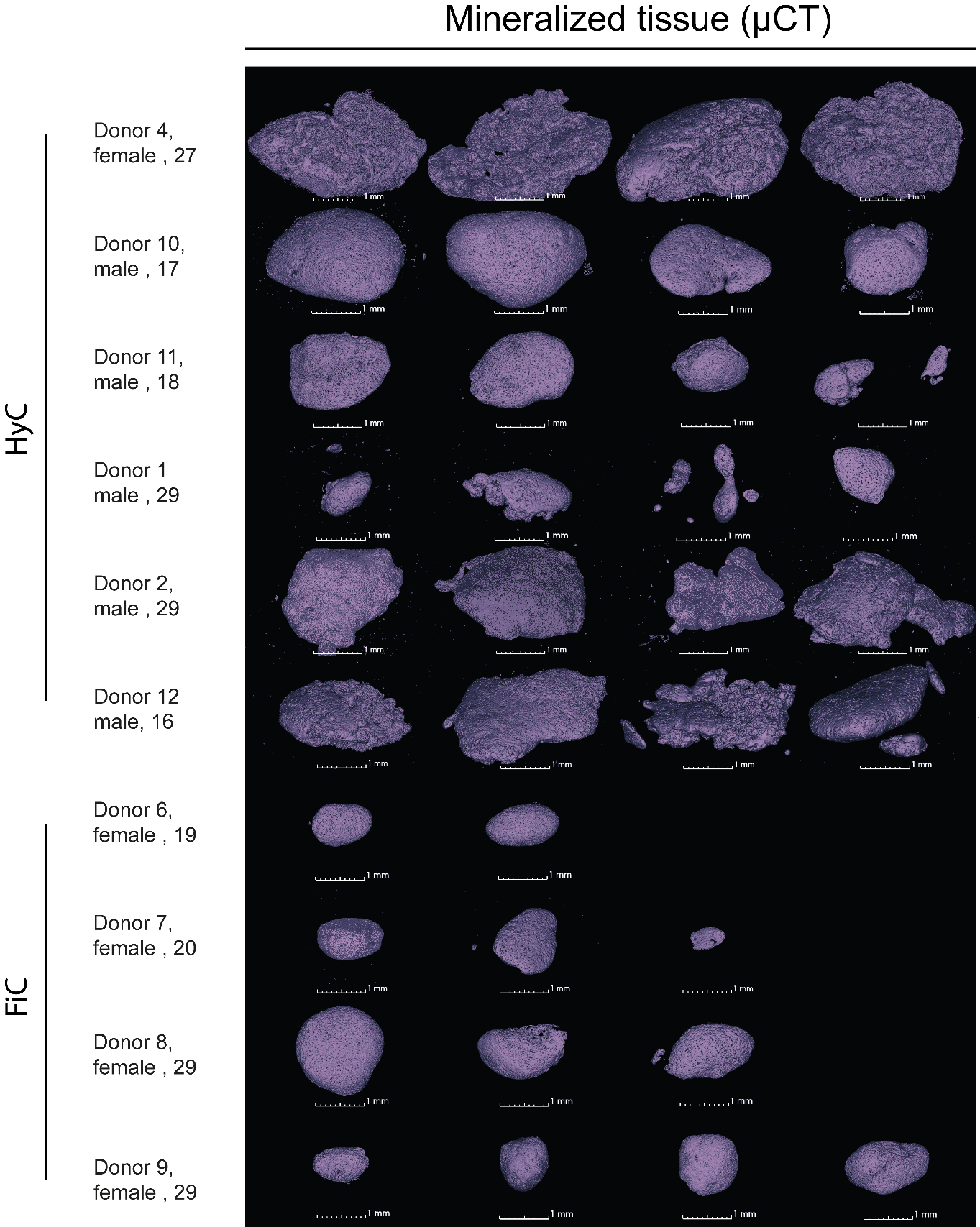


Fig. S7.

Extended dataset showing 3D representations of explants (n = 4) after 4 weeks of ectopic implantation, grouped per hypertrophic cartilage organoid (Hyc) and fibrocartilage organoid (FiC). Scalebar = 1 mm.


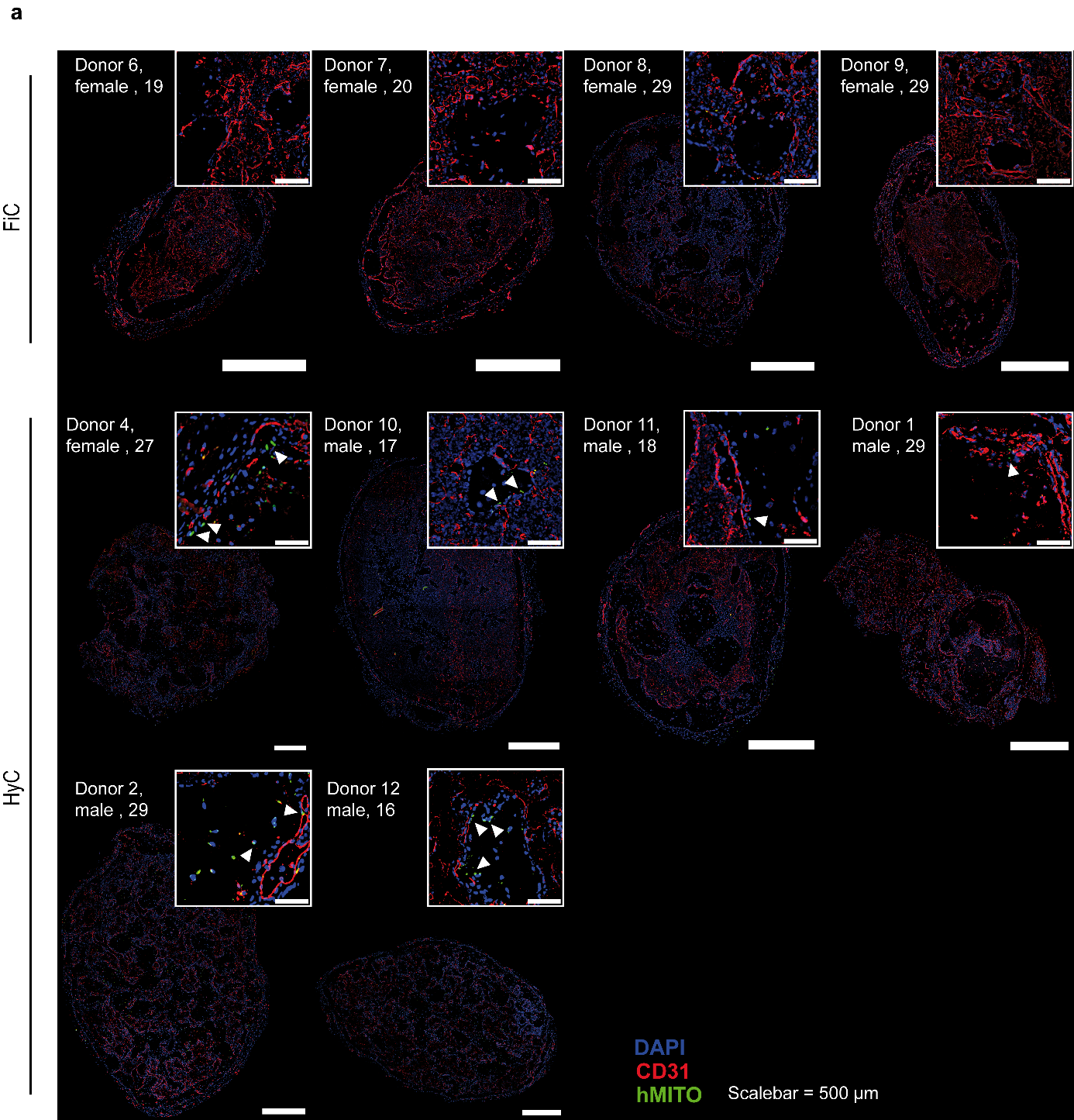
Fig. S8.

Extended dataset showing immuhistochemical staining for human mitochondria (green) and murine CD31 (red). Arrows point to human cells on the edge of vascularized regions. Scalebar = 500 µm or 50 µm in detail image.


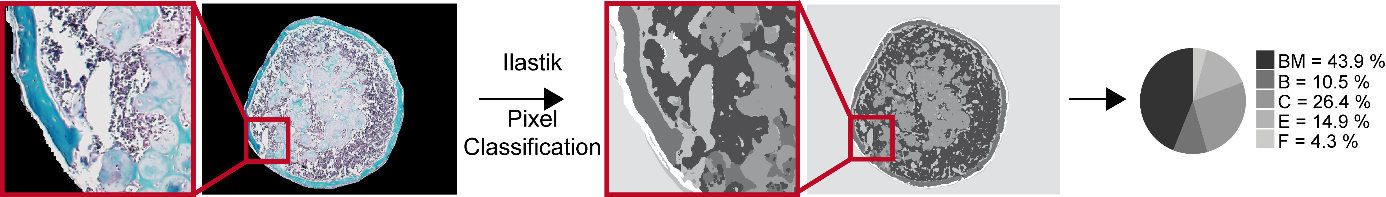
Fig. S9.

Explant tissue quantification following safranin O/FastGreen staining. BG = background, BM = bone marrow, B = bone, C = Cartilage, E = Empty, F = Fibrous.
